# A Bifurcated Proteoglycan Binding Small Molecule Carrier for siRNA Delivery

**DOI:** 10.1111/cbdd.12295

**Published:** 2014-05-13

**Authors:** Matt Gooding, Derick Adigbli, A W Edith Chan, Roberta J Melander, Alexander J MacRobert, David L Selwood

**Affiliations:** The Wolfson Institute for Biomedical Research, UCLGower Street, London, WC1E 6BT, UK; UCL Division of Surgery and Interventional Science, National Medical Laser CentreCharles Bell House, 67-73 Riding House Street, London, W1W 7EJ, UK; Centre for synthesis and chemical biology, University College DublinBenfield, Dublin 4, Ireland; Department of Chemistry, North Carolina State UniversityRaleigh, NC, 27695, USA

**Keywords:** biophysical chemistry, carbohydrate, chemical biology, lipid, nucleic acid, RNAi and antisense techniques

## Abstract

A wider application of siRNA- and miRNA- based therapeutics is restricted by the currently available delivery systems. We have designed a new type of small molecule carrier (SMoC) system for siRNA modeled to interact with cell surface proteoglycans. This bifurcated SMoC has similar affinity for the model proteoglycan heparin to an equivalent polyarginine peptide and exhibits significant mRNA knockdown of protein levels comparable to lipofectamine and the previously reported linear SMoC.

Small interfering RNAs (siRNA), which are capable of catalytically silencing specific genes via the RNA interference (RNAi) pathway, are attractive candidates for therapeutics for a wide range of diseases including genetic disorders and cancer. The direct application of unmodified siRNA to the target tissue has shown to be successful in clinical trials, for example an siRNA against a mutated form of keratin 6a to treat the skin condition pachyonychia congenita (TD101, Pachyonychia Congenita Project) 1, and several siRNA-based treatments for age-related macular degeneration 2, in which the naked siRNA is injected directly into the affected area. However, systemic delivery of siRNA, which is required for targeting internal organs and tumors, is more problematic as siRNA is readily degraded by endogenous nucleases and is rapidly cleared by the renal system. In addition, its large size and high anionic surface charge prohibits siRNA from crossing the plasma membrane and reaching the RNAi machinery in the cytoplasm 3.

To be systemically effective, siRNA must be formulated in such a way as to overcome these delivery barriers. siRNA encapsulated in nanoparticles which protect the siRNA from degradation and improve its half-life *in vivo* as well as aiding cell targeting and internalization is a popular strategy for increasing delivery efficiency 4. Several siRNA-containing nanoparticles have progressed to clinical trials, including TKM-PLK1 by Tekmira, currently in Phase II trials, which uses nucleic acid-lipid particles (LNPs) 5, 6 for patients with advanced solid tumors. Alnylam in partnership with Tekmira has developed two further LNP-based therapeutics, which are also currently in clinical trials: ALN-VSP in Phase II for liver cancer 7 and ALN-TTR01 for transthyretin-mediated amyloidosis which has completed Phase I trials 8. Another success for nanoparticles has been Calando's CALAA-01, in which siRNA complexed by a cationic cyclodextrin polymer has demonstrated for the first time in humans that systemically delivered siRNA can specifically silence genes 9, and is now in Phase I clinical trials. CALAA-01 consists of siRNA against the M2 subunit of ribonucleotide reductase (RRM2), which is involved in the proliferation of cancer cells, and targeted to cancer cells via a human transferrin protein targeting ligand. Patients with metastatic melanoma were exposed to the drug via 30 min iv infusion and resulted in significant reduction of RRM2 in the tumor cells. As in CALAA-01, polyethylene glycol (PEG) is frequently used to coat nanoparticles to prevent aggregation and binding to serum proteins as well as to evade detection by the immune system 10, 11. In addition, once the nanoparticles reach their target cells, they must penetrate the plasma membrane in order to allow the siRNA to access the RNA-induced silencing complex (RISC) located in the cytoplasm 12.

Cationic polymers such as polyethyleneimine 13, lipids 5, 6, 14, and cell-penetrating peptides (CPPs), including penetratin 15, polyarginine 16, and MPG 17 have been used to deliver siRNA *in vivo*, and form complexes via electrostatic interactions which neutralize the charge of the oligonucleotide and allow interaction with cell surface lipids and proteoglycans. As well as shielding the siRNA charge, these cationic materials have been found to promote cell entry of siRNA as they are effective at crossing cell membranes. Although the exact mechanism of cellular entry adopted by these polymers and peptides remains unclear, it is suggested that binding to cell surface proteoglycans such as heparan sulfate and chondroitin sulfate via their glycosaminoglycan (GAG) chains may be an initial internalization step, activating Rac1 and triggering subsequent rearrangement of the F-actin cytoskeleton leading to macropinocytosis initiation 18. Several studies have used heparin, a highly sulfated form of heparan sulfate, to estimate binding affinities of CPPs to cell surface GAGs, which were found to be in the micromolar to nanomolar range 19, 20, while a more recent study shows evidence that CPPs are able to form clusters with multiple heparin chains 21.

We previously described that the small molecule carrier (SMoC) class of compounds, which are small molecule mimics of penetratin, show potential as siRNA delivery agents 22, showing similar levels of gene silencing in IMR-90 cells to the commonly used transfection reagent Lipofectamine®. SMoCs possess cationic guanidine groups linked to a biphenyl backbone, forming amphipathic alpha-helix mimics which are capable of crossing cell membranes 23, 24. We postulated that the positive charges may be partially stabilized by *π*-cation interactions with the biphenyl backbone to promote electrostatic binding to siRNA and to anionic cell surface proteoglycans. The most successful SMoC compound for siRNA transfection described thus far is 4G-SMoC-SSPy **1**, (Figure 1, see Figure S1 for the naming convention for SMoCs). In the present study, we hypothesized that a bifurcated SMoC compound, 4G-BfSMoC-COOH, **2** would achieve the twin aims of cell surface proteoglycan binding and more flexible chemical synthesis. We observed that **2** retains the ability to complex and deliver siRNA, resulting in knockdown of the cell cycle gene *cdc7*. 4G*-*BfSMoC-COOH, **2** and other SMoCs show significant binding to heparin as a model for cell surface proteoglycans. **2** is easily synthesized and includes a central chemical ‘handle’ that will allow covalent attachment to other biomolecules.

**Figure 1:**
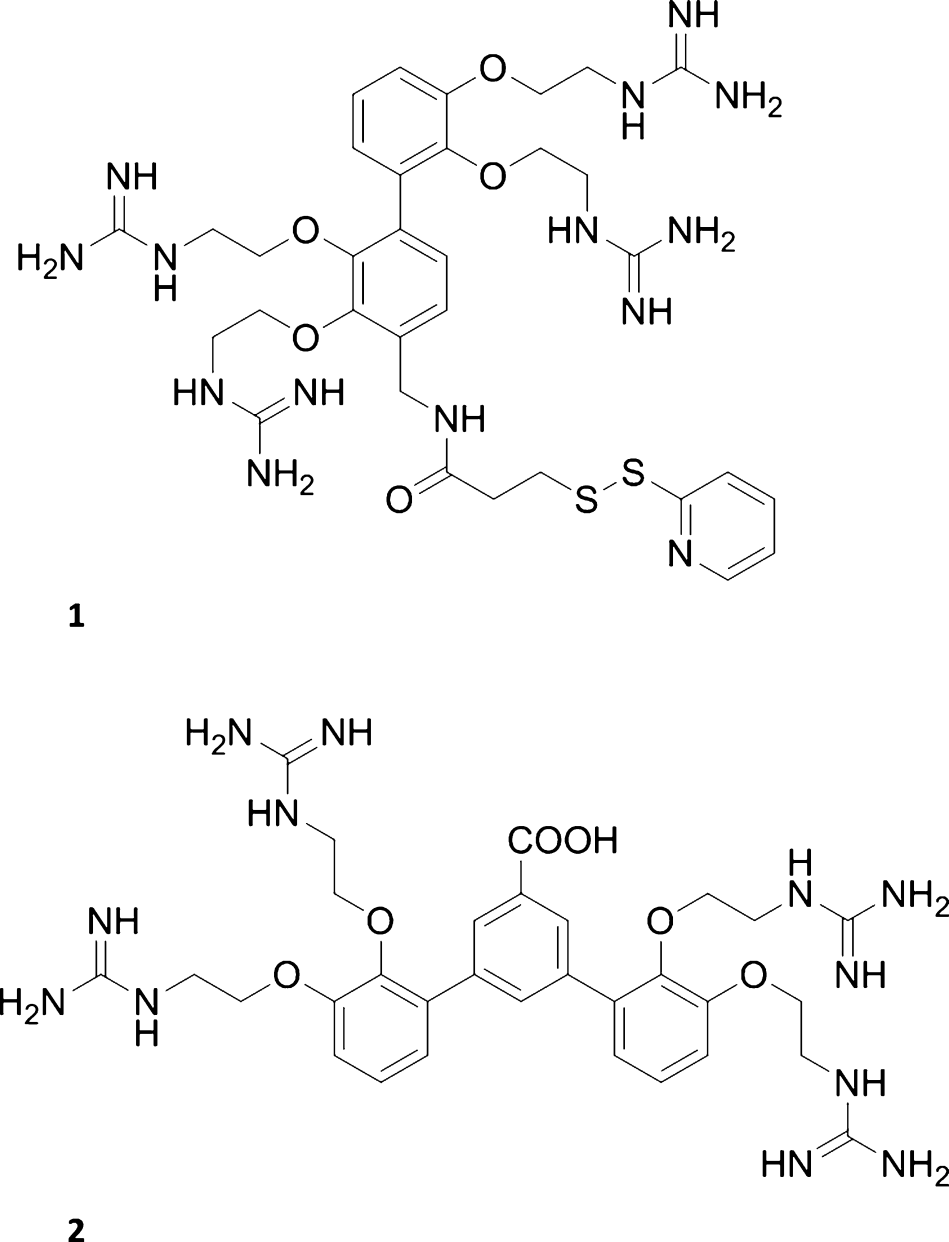
Chemical structures of 4G-SMoC-SSPy, 1 and 4G-BfSMoC-COOH, 2.

## Methods and Materials

Starting materials were either commercially available or synthesized according to methods reported in the literature. ^1^H and ^13^C NMR spectra were recorded on either a Bruker AMX-300 or a Bruker AMX-500 spectrometer. Chemical shifts are reported as ppm relative to TMS internal standard. Mass spectra were recorded on either a Fisons VG70-SE spectrometer (EI, FAB) or an Agilent 6100 Series LC-mass spectrometer (Wokingham, Berkshire, UK) using either a C-18 or C-4 column. Microwave reactions were carried out using a CEM Discover microwave. All compounds were purified to ≥ 95% as measured by LC-MS. Proteoglycan binding was recorded using an Agilent 1200 series HPLC system equipped with a diode array detector and HiTrap heparin agarose column. A complete list of ^1^H NMRs for compounds used in this study is given in the supplementary information (Table S1).

### Molecular modeling

Molecular modeling and dynamics were carried using Molecular Operating Environment (MOE) package (version 2011.10). The framework structures **3** and **4** were initially built in MOE (Chemical Computing Group, Montreal, Canada) using the ‘Molecular Builder’ with an extended conformation. All the guanidine groups are in +1 charge, reflecting the protonated state of the groups. Then, the initial structures were subjected to energy minimization using MMDD94X force field. The energy minimized structures of **3** and **4** were then carried through for dynamics simulations. All molecular dynamics simulations were conducted in a cube of water of depth of 10 Å using ‘Simulations-Dynamics’ in MOE. The protocol used in dynamics simulations is as follows: algorithm – Nosé-Poincaré-Andersen Hamiltonian equations of motion (NPA); temperature – constant at 300°K; mode – equilibration for 100 ps, forcefield – MMFF94X, cutoff at 8 Å. Conformations were collected in trajectory files every 0.5 ps, providing 200 conformers for each run for each molecule. The molecular dynamics simulations were also run in YASARA software 25 with similar results.

Data analysis, such as distance measurement, was carried out also in MOE using the graphics interface. The pairwise distances between the carbon atoms of the guanidine group from different phenyl rings in each molecule **3** or **4** were measured for each conformation. A histogram summarizing the data was calculated and plotted using Microsoft Excel.

Crystal structure information for heparin (PDB ID: 1HPN) and chondroitin sulfate (PDB ID: 1C4S) was obtained from the PDB and imported into DS Visualizer (Accelrys, San Diego, CA, USA) and the distance between sulfate residues measured between sulfur atoms.

### Synthesis

#### 3,5-bis(2,3-bis(2-(((benzyloxy)carbonyl)amino)ethoxy)phenyl)benzoic acid **6**

To a degassed solution of potassium (2,3-bis(2-(((benzyloxy)carbonyl)amino)-ethoxy)phenyl)trifluoroborate 22 (3.62g, 6.34 mmol) in isopropanol/water (2/1, 21 mL) was added PdCl_2_dppf.CH_2_Cl_2_ (123 mg, 0.15 mmol) and triethylamine (1.77 mL, 12.68 mmol) and the solution stirred for 2 min. 3,5-Diiodobenzoic acid, **5** (789.1 mg, 2.11 mmol) was added, and the reaction heated in a microwave reactor for 10 min at 75 °C. The reaction mixture was cooled, filtered, and 1 m HCl added (50 mL). The product was extracted with dichloromethane (3 × 25 mL), and the combined organic extracts were washed with brine (50 mL), dried over MgSO_4_, filtered, and concentrated under vacuum. The mixture was purified by flash column chromatography using a gradient of 0–50% ethyl acetate in cyclohexane and the title product (1.7 g, 80% yield) obtained as a colorless oil.

^1^H-NMR (500 MHz, CDCl_3_) *δ*: 3.27 (s, 4H, 2 × CH_2_), 3.48–3.74 (m, 8H, 4 × CH_2_), 4.11 (s, 4H, 2 × CH_2_), 4.97–5.10 (m, 8H, 4 × Ph-*CH*_*2*_*OCO*NH), 6.68–7.11 (m, 6H, 6 × ArH), 7.23–7.32 (m, 20H, 20 × Cbz-ArH), 7.66–8.29 (m, 3H, 3 × ArH).

^13^C-NMR (500 MHz, CDCl_3_) *δ*: 40.81 (CH_2_), 41.13 (CH_2_), 66.87 (Cbz CH_2_), 68.22 (CH_2_), 113.43, 123.04, 124.90, 128.20 (Cbz ArC), 128.51 (Cbz ArC), 135.03, 136.44, 138.37, 145.06, 152.02, 156.77 (C = O).

HRMS-ES (*m/z*): [M − H]^−^ calcd for C_59_H_57_N_4_O_14_, 1045.3871; found, 1045.3804.

#### 3,5-bis(2,3-bis(2-(((tert-butoxycarbonyl)guanidino)ethoxy)phenyl)-benzoic acid

To a solution of 3,5-bis(2,3-bis(2-(((benzyloxy)carbonyl)amino)ethoxy)phenyl)-benzoic acid **1** (813 mg, 0.7 mmol) in dichloromethane (20 mL) was added 30% hydrogen bromide in acetic acid (5 mL) dropwise and the mixture stirred at room temperature for 3 h. The reaction was diluted with dichloromethane (20 mL) and the product extracted with water (3 × 20 mL). The aqueous extracts were carefully dried under vacuum for several hours. The crude amine was then dissolved in methanol (20 mL), and *N*,*N′*-Di-Boc-1*H*-pyrazole-1-carboxamidine (922 mg, 3.15 mmol) and DIEA (1.22 mL, 7 mmol) were added. The reaction was stirred at room temperature for 24 h under nitrogen. The solvent was removed under reduced pressure and the residue redissolved in ethyl acetate (50 mL) and 1 m HCl added (50 mL). The layers were separated, and the aqueous layer washed with ethyl acetate (3 × 20 mL). The combined organic extracts were washed with brine (50 mL), dried over MgSO_4_, filtered, and concentrated under vacuum. The product was purified by flash column chromatography using a 0–25% ethyl acetate gradient in cyclohexane. The product was obtained as an off-white solid (651 mg, 63% yield).

^1^H-NMR (500 MHz, CDCl_3_) *δ*: 1.42–1.51 (m, 72H, 24 × Boc-CH_3_), 3.55 (m, 4H, 2 × CH_2_), 3.87 (m, 4H, 2 × CH_2_), 3.93 (m, 4H, 2 × CH_2_), 4.23 (m, 4H, 2 × CH_2_), 6.99 (m, 2H, 2 × ArH), 7.03 (m, 2H, 2 × ArH), 7.13 (m, 2H, 2 × ArH), 7.98 (m, 1H, ArH), 8.28 (m, 2H, 2 × ArH), 8.66 (m, 2H, 2 × NH), 8.81 (m, 2H, 2 × NH), 11.45 (bs, 1H, COOH).

^13^C-NMR (500 MHz, CDCl_3_) *δ*: 28.06 (Boc-CH_3_), 28.17 (Boc-CH_3_), 28.36 (Boc-CH_3_), 28.39 (Boc-CH_3_), 53.49 (CH_2_), 67.43 (CH_2_), 79.06 (Boc *C*CH_3_), 79.35 (Boc *C*CH_3_), 82.80 (Boc *C*CH_3_), 83.20 (Boc *C*CH_3_), 113.31, 123.34, 124.77, 129.78, 135.12, 138.33, 145.16, 152.11, 153.01, 156.43, 163.54, 169.33 (COOH).

HRMS-ES (*m/z*): [M+ H]^+^ calcd for C_71_H_107_N_12_O_22_, 1479.7423; found, 1479.7423.

#### 4G-BfSMoC-COOH, 3,5-bis(2,3-bis(2-(guanidinoethoxy))phenyl)-benzoic acid **2**

The tetra-Boc compound (30 mg, 0.02 mmol) was dissolved in DCM (2 mL), and TFA/*m*-cresol (95/5, 2 mL) was added and the mixture stirred for 4 h at room temperature. The solvent was removed under vacuum, and the residue washed three times with ether. The product was dissolved in water and lyophilized to form the tetra-TFA salt as a white solid (23 mg, 0.02 mmol, 100% yield).

^1^H NMR (600 MHz, D_2_O) *δ* 8.17 (d, *J *=* *1.6 Hz, 2H), 7.86 (d, *J *=* *1.1 Hz, 1H), 7.33–7.27 (m, 2H), 7.20 (dd, *J *=* *8.3, 1.2 Hz, 2H), 7.10 (dt, *J *=* *15.6, 7.8 Hz, 2H), 4.35–4.29 (m, 2H), 4.02–3.97 (m, 2H), 3.73 (t, *J *=* *4.9 Hz, 2H), 3.23–3.18 (m, 2H).

^13^C NMR (151 MHz, D_2_O) *δ* 171.06, 157.88, 157.21, 151.90, 144.44, 138.55, 135.89, 135.33, 130.64, 130.37, 126.24, 123.69, 114.53, 71.96, 67.55, 42.15, 41.61. Also visible peaks for trifluoroacetate, 163.98, 163.74, 163.51, 163.27, 119.86, 117.93, 116.00, 114.06.

HRMS-ES (*m/z*): [M+H]^+^ calcd for C_31_H_43_N_12_O_6_, 679.3429; found, 679.3428.

2, 3, 2′, 3′-tetra(2-guanidino-ethyloxy)-4-cyanobiphenyl, **8**, (4G-SMoC-CN).

To a solution of 2, 3, 2′, 3′-tetra(2-[*N, N*'-bis(tert-butoxycarbonyl)guanidino]-ethyloxy)-4-cyanobiphenyl **7** 22 (40 mg, 0.0289 mmol) in DCM (2 mL) was added a solution of TFA/m-cresol (95:5) (2 mL). The reaction mixture was stirred at room temperature for 3 h. Solvent was evaporated and the product precipitated from Et_2_O, washed three times with Et_2_O, dissolved in water, and lyophilized to give the title product (21 mg, 70%) as a white powder.

^1^H NMR (500 MHz, MeOH) *δ* 7.50 (d, *J *=* *8.0, 1H, aromatic), 7.20 (d, *J *=* *8.0, 2H, aromatic), 7.15 (dd, *J *=* *8.3, 1.6, 1H, aromatic), 6.91 (dd, *J *=* *7.5, 1.6, 1H, aromatic), 4.38 (t, *J *=* *5.0, 2H CH_2_), 4.24 (t, *J *=* *5.2, 2H, CH_2_), 4.00 (t, *J *=* *4.9, 2H, CH_2_), 3.92 (t, *J *=* *4.9, 2H, CH_2_), 3.68 (dt, *J *=* *9.8, 5.1, 4H, 2 × CH_2_), 3.28 (dd, *J *=* *9.6, 4.7, 4H, 2 × CH_2_).

HRMS *m/z* (ES^+^) 584.3186, calculated C_25_H_38_N_13_O_4_^+^ 584.3170.

### Gel shift assay

GAPDH siRNA (Thermo Scientific, Loughborough, UK; sequences used were sense 5′ UGG UUU ACA UGU UCC AAU AUU 3′, antisense 5′ Phosphate U AUU GGA ACA UGU AAA ACC UU 3′) was mixed in RNAse-free water with varying concentrations of either SMoC, PySSCH_2_CH_2_CO-RRRR-NH_2_, **9** (R4SSPy) or PySSCH_2_CH_2_CO-RRRRRRRR-NH_2_, **10**, (R8SSPy), (Peptide Synthetics, Ltd, UK) in the molar ratios of 1/1, 1/5, 1/10, 1/20 and 1/50. The final siRNA concentration was 17 *μ*m in 100 *μ*L of water (1.7 nmol) in each case. The complexes were incubated for 30 min at room temperature. The samples were diluted with 2× RNA loading dye (Fermentas, now Thermo Scientific) containing ethidium bromide (composition 95% formamide, 0.025% SDS, 0.025% bromophenol blue, 0.025% xylene cyanol FF, 0.025% ethidium bromide, 0.5 mm EDTA) and analyzed by gel electrophoresis on a 1% agarose gel prepared using 10× MOPS buffer [0.4 m MOPS (pH 7), 0.1 m sodium acetate, 0.01 m EDTA (pH 8)], loading 25 *μ*L of sample into each well. The gel was run at 5V/cm till the blue dye had migrated 2/3 of the gel. The gels were analyzed by UV illumination and the ethidium bromide bands quantified using the ImageJ software (NIH, Bethesda, MD, USA). The EtBr intensities were plotted, a dose–response curve fitted and EC_50_ values calculated using the Origin software (OriginLab, Northampton, MD, USA).

### Cell culture

IMR-90 (ATCC# CCL-186) cells were obtained from LGC Standards, UK, at population doubling (PD) 12. Cells were cultured in Dulbecco's modified Eagles Medium (DMEM) (Invitrogen, Life Technologies, Paisley, UK) supplemented with 10% Fetal Bovine Serum (Invitrogen) and 2 mm l-glutamine (Invitrogen) at 37 °C and 5% CO_2_. 4T1 cells were purchased from Caliper Life Sciences (Hopkinton, MA, USA). MCF-7 cells were obtained from the European Collection of Animal Cell Cultures, Porton Down, UK.

### Confocal microscopy *in vitro*

Cell preparation: 1 × 10^4^ 4T1 (murine) and MCF-7 (human) breast cancer cells were seeded in 35 mm diameter glass-bottomed fluorodishes (WPI, Hitchin, Hertfordshire, UK). These were incubated overnight to encourage adherence and optimal subconfluent cell spreading for imaging. 4T1 cells were incubated with fresh RPMI-1640 Medium with l-glutamine and sodium bicarbonate (Sigma-Aldrich, Poole, Dorset, UK) supplemented with 10% (v/v) fetal calf serum (FCS) (Gibco, Life Technologies, Paisley, UK, Invitrogen); MCF-7 cells were incubated with fresh DMEM containing 0.365 g/L l-glutamine, 4.5 g/L glucose (BioWhittaker, Verviers, Belgium), supplemented with 10% (v/v) FCS (Sigma-Aldrich), 100 U/mL penicillin and 100 *μ*g/mL streptomycin at 37 °C in a humidified 5% CO_2_ incubator.

Confocal: Cells were observed using an inverted Olympus Fluoview 1000 confocal laser-scanning microscope to determine intracellular localization of the oligonucleotide. Fluorescence confocal images were obtained using a 60× NA:1.35 oil immersion objective lens (Olympus Fluoview FV1000; Olympus, Southend-on-Sea, UK). Original image files were analyzed using Image J software. Analysis of the labeled siRNA ± 4G-BfSMoC-COOH, **2** was performed using image J to quantify the mean intracellular signal intensity compared to the background level of fluorescence. A one-way anova with Tukey's multiple comparison test was used to assess statistical significance. Appropriate controls were used throughot.

#### RNA oligonucleotide, lipofectamine, and 4G-BfSMoC-COOH, **2** preparation

The intracellular localization of Alexa Fluor® Red Fluorescent oligonucleotide (Invitrogen) supplied as a 20 *μ*m stock of Alexa Fluor® 555-labeled, double-stranded RNA oligomer in 100 mm KOAc, 30 mm HEPES-KOH, pH 7.4, and 2 mm MgOAc (annealing buffer) was determined through confocal fluorescence microscopy. Lipofectamine® RNAiMAX (Invitrogen). The oligonucleotide was diluted to a final concentration of 1 *μ*m either alone or in combination with 10 *μ*m 4G-BfSMoC-COOH, **2**. The initial dilutions were in phosphate buffered saline (PBS) (Lonza, Slough, UK). The oligonucleotide/4G-BfSMoC-COOH, **2** mixture was allowed to complex for 15 min at room temperature. Final dilutions were made in the appropriate cell medium. Either cell line was subsequently incubated with media (alone), oligonucleotide(alone), 4G-BfSMoC-COOH, **2** (alone), or oligonucleotide/4G-BfSMoC-COOH, **2** for 24 h. Lipofectamine controls were transfected according to manufacturer's instructions to a final concentration of 3.75 *μ*L/mL + oligonucleotide. Cells were washed twice with PBS and treated with phenol-red-free media prior to confocal imaging.

### siRNA transfection

Solutions were made up in Opti-MEM® (Invitrogen); 100 nm *cdc7* siRNA (sense 5′-GCU CAG CAG GAA AGG UGU UUU-3′, antisense 5′-AAC ACC UUU CCU GCU GAG CUU-3′; Applied Biosystems, Warrington, UK), 100 nm Accell Non-targeting siRNA 1 negative control siRNA (Thermo Scientific), 8 *μ*L/mL Lipofectamine LTX reagent (Invitrogen), 0.5 mm SMoC. SMoC/siRNA, and Lipofectamine/siRNA complexes were made by mixing equal volumes of the required solutions and incubating for 30 min at room temperature.

Cells were plated in 6-well plates at a density of 5 × 10^5^ cells/well in 1 mL DMEM containing 10% FCS and 2 mm glutamine and incubated at 37 °C under 5% CO_2_ for 24 h. The media was removed and replaced with 1.6 mL Opti-MEM® (Invitrogen). Four hundred microliters of SMoC/siRNA or Lipofectamine/siRNA complexes was added and the cells incubated for a further 72 h. The final concentration of SMoC was 50 *μ*m and based on the dose–response analysis reported previously 22.The cells were washed with PBS and trypsinized and the pellet stored at −80 °C.

### RNA purification and qRT-PCR

RNA was extracted from the cell pellets using the PureLink RNA Mini kit (Invitrogen) using the protocol provided by the manufacturer. RNA was quantified using a Nanodrop spectrophotometer, and 40 ng of RNA was mixed with Superscript III RT/Platinum *Taq* Mix and SYBR Green Reaction Mix from the Supersript III Platinum SYBR Green One-Step qRT-PCR kit (Invitrogen) according with the manufacturer's protocol. PCR conditions were 50 °C for 3 min, 95 °C for 10 min (one cycle), 95 °C for 15 seconds, 47 °C for 30 seconds, 60 °C for 30 seconds (45 cycles), 95 °C for 15 seconds, 60 °C for 15 seconds, ramp to 95 °C over 20 min (one cycle). Relative quantification data were obtained using the comparative *C*_t_ method with Realplex software according to the manufacturer's protocol (Eppendorf, Hamburg, Germany). c*dc7* RNA was measured using the following primers: forward primer: 5′-AAC TTG CAG GTG TTA AAA AAG-3′: reverse primer: 5′-TGA AAG TGC CTT CTC CAA T-3′. The following primers were used to measure GAPDH: forward primer: 5′-TCA ACT ACA TGG TTT ACA TGT TC-3′: reverse primer: 5′-GAT CTC GCT CCT GGA AGA T-3′. The signal obtained from the c*dc7* primers was normalized using the signal from the GAPDH primers.

### Proteoglycan binding

The test SMoCs and the marker oligoarginine peptides R4SSPy, **9** and R8SSPy, **10** were applied to a heparin agarose column (HiTrap, GE-Healthcare Life Sciences, UK) (1 mL) and eluted with a gradient of sodium chloride from 500 to 1000 mm. The absorbance at 214 nm (CONH) was recorded and exported to Origin 9.0. The signals were normalized and replotted as shown in the Figure. HIVtat, **11**, (sequence YGRKKRRQRRR) was obtained from Sigma.

### Statistical analysis

Groups were compared using an unpaired two-sample Student's *t*-test. A value of *P* < 0.05 was considered significant.

## Results

### Design of a bifurcated SMoC derivative

**Figure 2:**
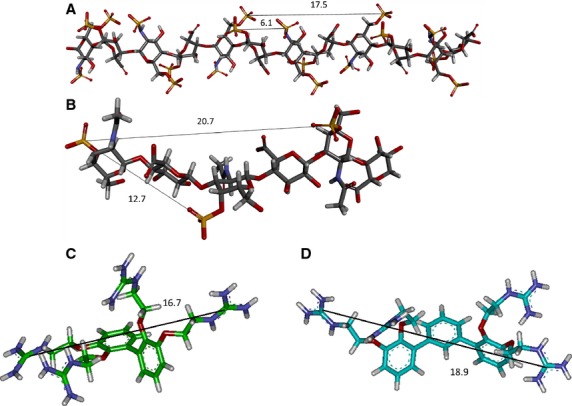
Modeling a new SMoC analogue for binding to cell surface glycosaminoglycans. (A) Examination of the crystal structure of heparin. Clusters of three sulfate residues are observed, occurring on alternating sides of the sugar chain. Representative distances between residues in the same cluster, and between residues of adjacent clusters on the same side of the chain are shown in Å. (B) Chondroitin sulfate crystal structure obtained from the PDB. The distances between sulfate ions are marked in Å. (C) A representative snapshot taken from the molecular dynamics simulation of 3. The average distance between the furthest guanidine groups is indicated in Å. (D) A snapshot from the molecular dynamics simulation of 4. The average distance between the furthest guanidine groups is indicated in Å.

As binding to cell surface GAGs such as heparan sulfate and chondroitin sulfate is thought to play a role in CPP internalization, the active transfection compound **1** (Table1) was studied via molecular modeling and dynamics for its ability to bind these proteoglycans. The structures of chondroitin sulfate and heparin, a highly sulfated form of heparan sulfate, were obtained from the Protein Databank, and the distances between sulfate residues were measured (Figure 2A,B). Examination of the crystal structure of heparin (pdb: 1HPN) revealed that the sulfate residues were positioned in ‘clusters’ of three on alternating sides of the sugar chain. The distance between residues within each cluster varies between 6.1 and 7.8 Å with an average distance of 6.6 Å, whereas the distance between clusters on the same side of the chain averages of 17.5 Å (Figure 2A). Due to flexibility of the chain, it is possible that in nature, the distances between sulfate residues may vary considerably from the crystal structure. For the crystal structure obtained for chondroitin sulfate (pdb: 1C4S), the distance between sulfate groups was measured as 12.7 Å (Figure 2B), with sulfate groups positioned on alternating sides of the sugar chain. As the SMoC guanidine groups may not be able to access sugars on opposite sides of the chain, the distance between sulfates on the same side was also measured and found to be approximately 20.7 Å.

**Table 1:** Proteoglycan binding: retention times of arginine rich peptides and SMoCs on a heparin column

Compound	Structure	Short name	Guanidines	Retention time	Reference for compound
**9**	PySSCH_2_CH_2_CO-RRRR-NH_2_	R4SSPy	4	4.4	22
**10**	PySSCH_2_CH_2_CO-RRRRRRRR-NH_2_	R8SSPy	8	19.17	22
**11**	YGRKKRRQRRR	HIV-1 tat	6	15.2	This study
**1**	See Figure 1	4G-SMoC-SSPy	4	8	23
**2**	See Figure 1	4G-BfSMoC-COOH	4	4.69	This study
**12**	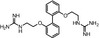	2G-SMoC	2	3.15	22
**13**	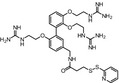	3G-SMoC-SSPy	3	4.6	24
**8**	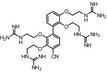	4G-SMoC-CN	4	7.4	This study
**14**	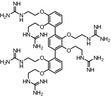	6G-SMoC	6	17.12	22

**Figure 3:**
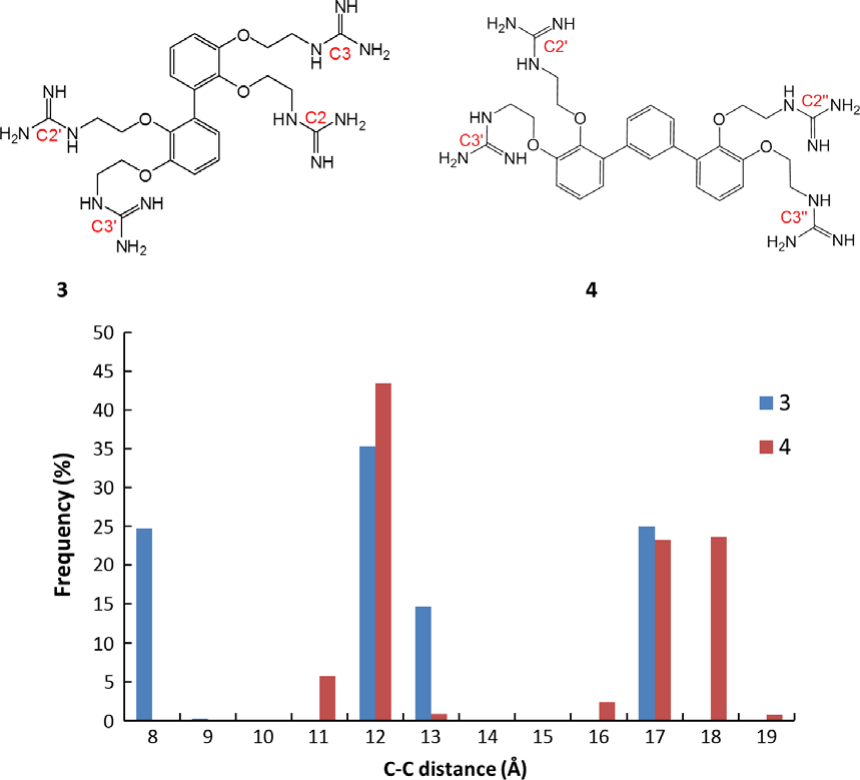
Frequency of distances: between the two carbon atoms of the guanidine groups attached to different phenyl rings for structures 3 and 4. The carbon atoms are labeled in red in the diagram.

To gauge the interaction between our molecules and heparin and chondroitin sulfate, the pairwise distances between the carbon atoms of the guanidine group from different phenyl rings for each conformation observed during molecular dynamics simulation of structure **3** were measured; that is, four distances were measured: between C2-C2′, C2-C3′, C3-C2′, and C3-C3′ as labeled in Figure 3. The results are plotted as a histogram showing the frequency of the C-C distances. More than 75% of the time, the C-C distances are above 11 Å. The most frequently occurring distances between two carbons (35%) are between 11 and 12 Å, mainly between groups C3-C2′ and C2-C3′. The longest distance is averaged around 16.48 Å, contributed mainly from groups C3-C3′. This distance is sufficient for binding to heparin sulfates in the same cluster, as well as binding to chondroitin sulfates on opposite sides of the sugar chain. However, to optimize binding to these GAGs, the distance between guanidine residues should be slightly increased in order that it is possible to bridge the gap between heparin sulfate clusters, as heparan sulfate is less sulfated than heparin, and the sulfate groups may be more sparsely arranged. Furthermore, macropinocytosis is initiated by clustering of GAG chains attached to different proteoglycan molecules 26. Therefore, increased spacing between SMoC guanidine groups may allow for binding of sulfates on different GAG chains.

To extend the distance between guanidine groups in the SMoC structure, we decided to investigate the incorporation of an additional phenyl ring to the original structure, to produce a bifurcated structure, **4** (Figure 3). Again molecular dynamics was carried out for this molecule. We predicted that the ring should be added at the same location as the biphenyl bond to preserve the *π*-cation stabilization of the guanidine charges 22. The pairwise distances between the carbon atoms of the guanidine group from different phenyl rings for each conformation observed during molecular dynamics simulation of structure **4** were measured; that is, four distances were measured: between C2′-C2″, C2′-C3″, C3′-C2″, and C3′-C3″. From Figure 3, almost half of the time (50%), the distances between any 2 guanidine carbon atoms are larger than 15 Å; and 25% of the distances are above 17 Å, contributed mainly from groups C3′-C3″. Therefore, compound **2** is sufficient for binding to adjacent sulfate clusters in heparin and may also bind heparan sulfate in a different way to **1** at the cell surface. The additional distance between guanidines may also allow binding of additional sulfate groups on chondroitin sulfate, as well as increasing the possibility of promoting clustering of GAG chains.

### Synthesis of 4G-BfSMoC-COOH

**Figure 4:**
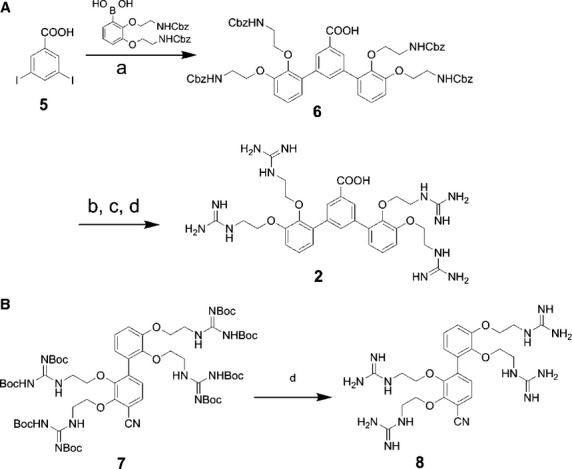
Synthesis of 4G-BfSMoC-COOH (A) and the marker compound 4G-SMoC-CN (B). Reagents: (a) PdCl_2_dppf. CH_2_Cl_2_, Et_3_N, iPrOH/H_2_O, MW 80 °C (80%), 10 min; (b) 30% HBr in acetic acid, DCM, rt; (c) *N*,*N′*-Di-Boc-1*H*-pyrazole-1-carboxamidine, DIEA, DCM, room temperature, 17 h (63% over 2 steps); (d) TFA/TIPS/H_2_O, rt, 4 h (100%).

The synthesis of the new SMoC partly followed the synthetic route previously described for the biphenyl 4G-SMoC-SSPy, **1** 22 (Figure 4A). The additional phenyl ring was added via 3,5-diiodobenzoic acid, **5** a commercially available compound that also contains an acid for use as a linker group to attach targeting ligands. The synthesis made use of the trifluoroborate salt described previously 22, which had been synthesized in bulk, in order to introduce the amine functionality for adding guanidine groups. The terphenyl structure was created using a Suzuki-Miyaura palladium coupling reaction under microwave irradiation at 80 °C with the trifluoroborate salt, producing the coupled product **6** in 80% yield. The guanidinylation step proceeded in the presence of the unprotected acid group using the pyrazole guanidinylation reagent in 63% yield, demonstrating that the acid does not react with the guanidinylation reagent. The guanidines were deprotected with TFA to yield the final product **2**. The 4-cyano intermediate **7** described previously 22 was utilized for the synthesis of the marker molecule **8** by deprotection with trifluoroacetic acid/TIPS (Figure 4B).

### siRNA complexation

**Figure 5:**
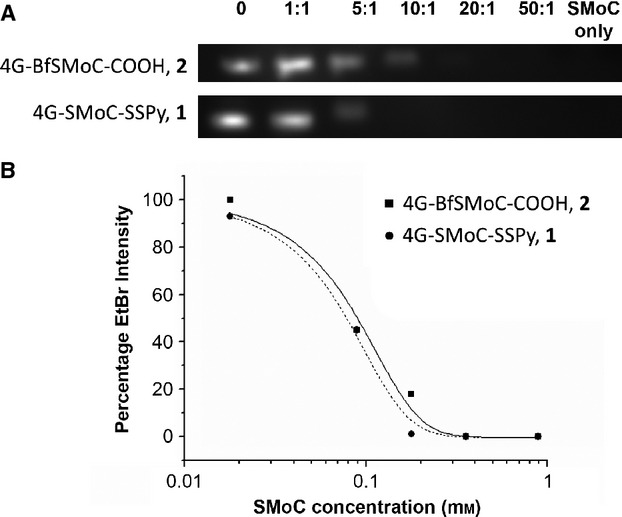
4G-BfSMoC-COOH, 2 and 4G-SMoC-SSPy, 1 have similar affinity for siRNA. (A) GAPDH siRNA (17 *μ*m) was mixed with 1 or 2 at the molar ratios shown with a loading dye containing ethidium bromide and run on a 1% agarose gel using a 0.4 m MOPS buffer (pH 7.0) containing 0.1 m sodium acetate and 0.01 m EDTA. The gel was visualized under UV illumination. (B) The ethidium bromide intensities as a function of SMoC concentration were fitted in Origin. The EC_50_ value and Hill coefficient (*P*) were calculated from the binding curve.

To confirm that the new SMoC retains the ability to bind siRNA, the binding affinity was assessed using a gel shift assay and compared to our previously tested 4G-SMoC-SSPy, **1** (Figure 5A). The intensity of the siRNA bands detected using ethidium bromide is reduced in a dose-dependent fashion with the addition of the SMoCs due to neutralization of the negative charge, resulting in immobilization of the siRNA on the gel. The EC_50_ calculated for **2** was 0.07 mm (Figure 5B), which is equivalent to **1**. This shows that **2** maintains the same level of siRNA-binding capabilities as its predecessor, the maintenance of this interaction is not obvious as the distances and angles between the phenyl groups displaying the guanidines is altered; in addition, the less electron-rich central phenyl ring might alter the strength of *π*-cation interactions.

### siRNA transfection observed using fluorescent siRNA oligomers

**Figure 6:**
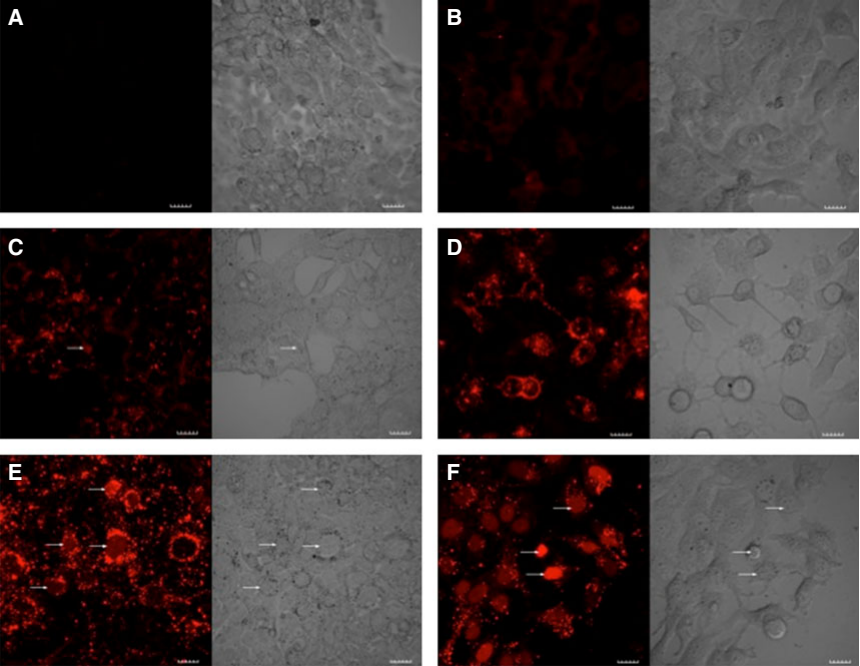
Cellular uptake of fluorescent siRNA. Confocal microscopy of cells treated with AlexaFluor® Red Fluorescent Oligonucleotide ± 4G-BfSMoC-COOH, 2. Cells were treated with oligonucleotide-fluorophore alone (A) 4T1 and (B) MCF7; or treated with oligonucleotide-fluorophore plus 4G-Bf-SMoC-COOH (C) 4T1 and (D) MCF-7 or oligonucleotide plus lipofectamine (E) 4T1 and (F) MCF-7 (scale = 20 μm). Quantitative analysis of the relative cellular uptake demonstrates that addition of the 4G-BfSMoC-COOH, 2 resulted in 4.9-fold (*P* < 0.01) increased uptake of the oligonucleotide-fluorophore (siRNA) in the 4T1 cells; and a 2.6-fold (*P* < 0.01) increase in the MCF7 cell line. There was evidence of nuclear uptake in the 4T1 cell line (Figure 6C, white arrow). In cells treated with Lipofectamine plus siRNA, we observed 14.4-fold and 3.3-fold increase uptake in 4T1 and MCF-7 cell lines, respectively. There was significant nuclear uptake in both cell lines following treatment with Lipofectamine (Figure 6D and E, white arrows). The differing level of background uptake (siRNA alone) is likely due to the differing characteristics of the two cell lines including doubling times and uptake kinetics.

To examine the intracellular localization of the 2-enhanced siRNA import, we utilized confocal microscopy with fluorescently labeled siRNA (Figure 6). Quantitative analysis of the relative uptake demonstrates that addition of (10 *μ*m) 4G-BfSMoC-COOH, **2** resulted in 4.9-fold (*P* < 0.01) increased uptake of the oligonucleotide-fluorophore in the 4T1 cells; and a 2.6-fold (*P* < 0.01) increase in the MCF7 cell line compared to siRNA alone. There was undetectable ‘background’ uptake of fluorescently labeled siRNA alone in the 4T1 cell line (Figure 6A) and non-significant uptake in the MCF-7 cells (Figure 6C). This indicates that there is minimal uptake and retention of uncomplexed oligonucleotide-fluorophore by MCF-7 cells at 24 h. The difference between the two cell lines indicates that this likely to be a cell specific process. Negative controls (without oligonucleotide-fluorophore) showed no fluorescent signal; thus, this background effect is unlikely to be artifact but rather a feature of the uptake kinetics of the specific cell lines. Positive controls using lipofectamine demonstrated 14.4-fold (*P* < 0.01) and 3.3-fold (*P* < 0.01) increased uptake compared to siRNA alone in 4T1 and MCF-7 cell lines, respectively. In both cases, there was significant nuclear uptake at 24 h (Figure 6E,F). Other compounds listed in Table1 either were inactive as reported previously 22 or in the case of the 4G-SMoC-CN, **8** were utilized only as a control.

### siRNA transfection using 4G-BfSMoC-COOH, **2**

**Figure 7:**
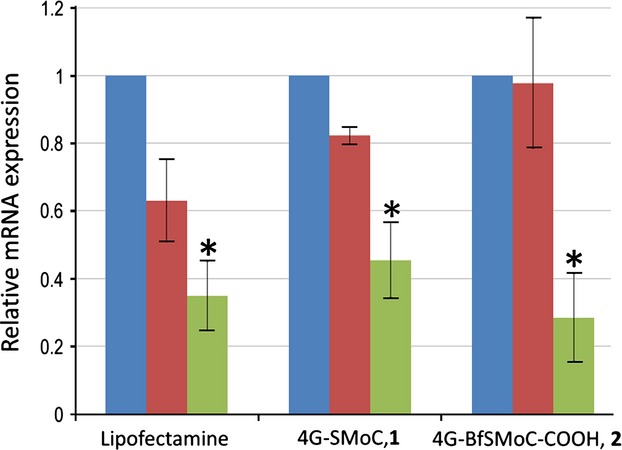
mRNA expression levels in cells treated with 4G-BfSMoC-COOH:siRNA complexes. qRT-PCR data showing *cdc7*mRNA expression in either untreated IMR-90 cells (blue) or cells treated with Lipofectamine®, 1, or 2 complexed to control (red) or *cdc7* (green) siRNA. Experiments were conducted in triplicate. *, *P* < 0.05 compared to control siRNA.

Both the original 4G-SMoC-SSPy, **1** and the new 4G-BfSMoC-COOH, **2** were used to transfect either *cdc7* siRNA or a negative control siRNA into IMR-90 primary human fibroblasts at a final SMoC concentration of 50 *μ*m as described previously 22. The commonly used transfection reagent Lipofectamine® was also used as a positive control, and the mRNA knockdown was determined by quantitative real-time PCR (Figure 7). The new 4G-BfSMoC-COOH achieved a knockdown of *cdc7* mRNA of 71% compared to the negative control siRNA, whereas the original 4G-SMoC-SSPy produced a 45% knockdown and was identical to Lipofectamine®, as predicted by our earlier study. However, the difference between the bifurcated SMoC and the original compound was not statistically significant in this study.

### Proteoglycan binding of SMoCs compared to polyarginine peptides and HIV-**1** tat_47–57_

**Figure 8:**
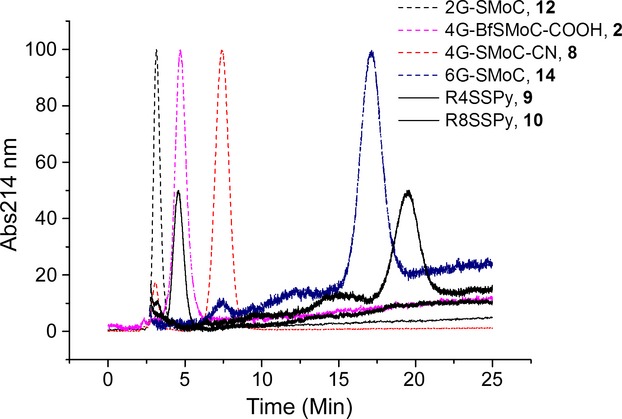
Relative binding to a heparin surface. The SMoCs shown are applied to a HiTrap heparin column in PBS pH 7.4 and eluted with an increasing gradient of NaCl 0.5–1 m. Polyarginine peptides are added as references (half height peaks). A full list of the retention times is shown in Table1.

To determine the relative proteoglycan binding of SMoCs, we utilized heparin bound to agarose beads and monitored the retention time when a gradient of sodium chloride (500–1000 mm) was applied to the column. Polyarginine peptides R4SSPy, **9** and R8SSPy, **10** were applied as markers. Selected overlaid traces are shown in Figure 8 and the full dataset in Table1. The affinities of 2G-SMoC, **12** a 4G-SMoC marker compound bearing a 1-CN group, **8**, and 6G-SMoC, **14** 22 increase in proportion to the number of guanidine groups. 4G-BfSMoC-COOH, **2** shows similar affinity to the linear 3G-SMoC-SSPy, **13** and somewhat less than the 4G-SMoCSSPy, **1** perhaps due to the carboxylic acid group which is negatively charged at physiological pH. Affinity to heparin was insensitive to the inclusion of the disulfide pyridyl group (see entries for 4G-SMoC-CN, **8** and 4G-SMoC-SSPy, **1**. HIV-1 tat_47–57_, **11** showed less affinity than the R8SSPy polyarginine peptide, **10** and similar affinity to 6G-SMoC **14**. Attempts to determine the proteoglycan binding of lipofectamine were unsuccessful using this technique. Proteoglycan binding cannot be assessed as Lipofectamine is a cationic liposome-based reagent, comprising a 3:1 (w / w) liposome formulation of the polycationic lipid 2,3-dioleyloxy-*N*-[2(sperminecarboxamido)ethyl]-*N*,*N*-dimethyl-1-propanaminium trifluoroacetate (DOSPA) and the neutral lipid dioleoyl phosphatidylethanolamine (DOPE) in membrane-filtered water. The only UV active component is expected to be DOSPA with a single weak amide UV absorbance expected at 214 nm. However, using our standard conditions and monitoring at 214 nm, we observed only a peak at the solvent front even with 10 μg of Lipofectamine injected (data not shown).

## Discussion

The development of a delivery vector that is able to complex and protect siRNA *in vivo* as well as achieve efficient and targeted cellular internalization would be a major advancement for the therapeutic use of siRNA. Multicomponent siRNA delivery nanoparticles may consist of layers of different materials, such as cationic polymers for siRNA complexation, PEG to increase serum stability, and targeting ligands to confer cell specificity. An efficient siRNA transfection vector should tightly package the siRNA, bind to the cell surface so as to induce macropinocytosis, and finally escape from the endosome into the cytoplasm in order for the siRNA to act on the RNAi machinery and silence the target gene. In this study, we have examined the ability of the SMoC class of transporter to achieve the earlier stages of this process – namely siRNA complexation and cell surface proteoglycan binding.

We previously showed that the protein transduction compound 4G-SMoC-SSPy, **1** was also active as a siRNA delivery vehicle. It was demonstrated that SMoCs were capable of packaging siRNA via electrostatic complexation through the guanidine side chains, thus neutralizing the siRNA negative charges. We also postulated that the biphenyl ring system of 4G-SMoC-SSPy formed *π*-cation interactions with the guanidine groups, therefore shielding the positive charges during membrane penetration, as predicted for penetratin. In the present study, we develop the structure of the original SMoC to produce a siRNA delivery agent that is able to bind sulfate groups on adjacent cell surface GAGs, is simpler to synthesize and contains a chemical ‘handle’ for potential conjugation.

The new 4G-BfSMoC-COOH, **2** described has been molecularly designed to explore binding to the common GAGs heparan sulfate and chondroitin sulfate, which have been shown to interact with cell-penetrating peptides as a precursor to internalization. In a model system utilizing the more highly sulfated proteoglycan heparin, we observed that the SMoCs bound with similar affinity to the reference tetra and octa-arginine **10**, **11**, and HIV-1 tat peptides. 4G-BfSMoC-COOH, **2** did not show increased binding over linear SMoCs despite the clear indication from the modeling studies that longer distances might be beneficial for binding and the molecular dynamics studies indicated that the larger structures were able to adopt more extended conformations. We should be cautious, however, in that cellular binding may differ from our model heparin system. Several studies have suggested that the binding of GAG by CPPs plays a role in cell penetration, with subsequent receptor clustering leading to reorganization of the actin cytoskeleton as a precursor to macropinocytosis 18, 20, 27–30. Heparin has been used in binding assays to determine GAG-binding affinities for several CPPs, which are found to bind in the low micromolar to nanomolar range 19, probably via bidentate hydrogen bonds between arginine residues and sulfate groups. Recently a report from the Dowdy laboratory challenged the importance of GAG binding for CPP internalization 31, and the uptake of other peptides has been shown to be independent of GAG binding 32, 33. Further investigations will be required to ascertain the exact influence of GAG binding on the SMoC class of biomolecular transporter. The new SMoC described here was easily synthesized, with the trifluoroborate salt used in a Suzuki-Miyaura bicoupling step to form the terphenyl structure. The success of this coupling step demonstrates that the trifluoroborate salt may be used to generate larger branched SMoC analogues from simple starting materials. The bifurcated SMoC does not show increased uptake compared to the original 4G-SMoC-SSPy, **1** reagent or to Lipofectamine®; however, it does demonstrate that the disulfide pyridyl group, and therefore potential reaction with cell surface thiols, is not required for successful siRNA transfection. Further optimization of this system is in progress.

## Supporting Information

Figure S1 Naming convention for SMoCs. **Table S1** Following a request from a reviewer we have included all the ^1^H NMRs for the compounds used in this study.
