# Supplementary material for: A Bifurcated Proteoglycan Binding Small Molecule Carrier for siRNA Delivery
Source: Chem Biol Drug Des. 2014 May 13;84(1):24–35. doi: 10.1111/cbdd.12295 (PMC4286013; doi:10.1111/cbdd.12295)
Supplement: Figure S1 — Naming convention for SMoCs. Table S1 Following a request from a reviewer we have included all the 1H NMRs for the compounds used in this study. [file cbdd0084-0024-sd1.docx]

**Supplementary information**

**A Bifurcated Proteoglycan Binding Small Molecule Carrier for siRNA delivery**

**Authors**

Matt Gooding, Derick Adigbli, A. W. Edith Chan, Roberta J. Melander, Alexander J.MacRobert and David L. Selwood.

**Supplementary Figure S1.** Naming convention for SMoCs


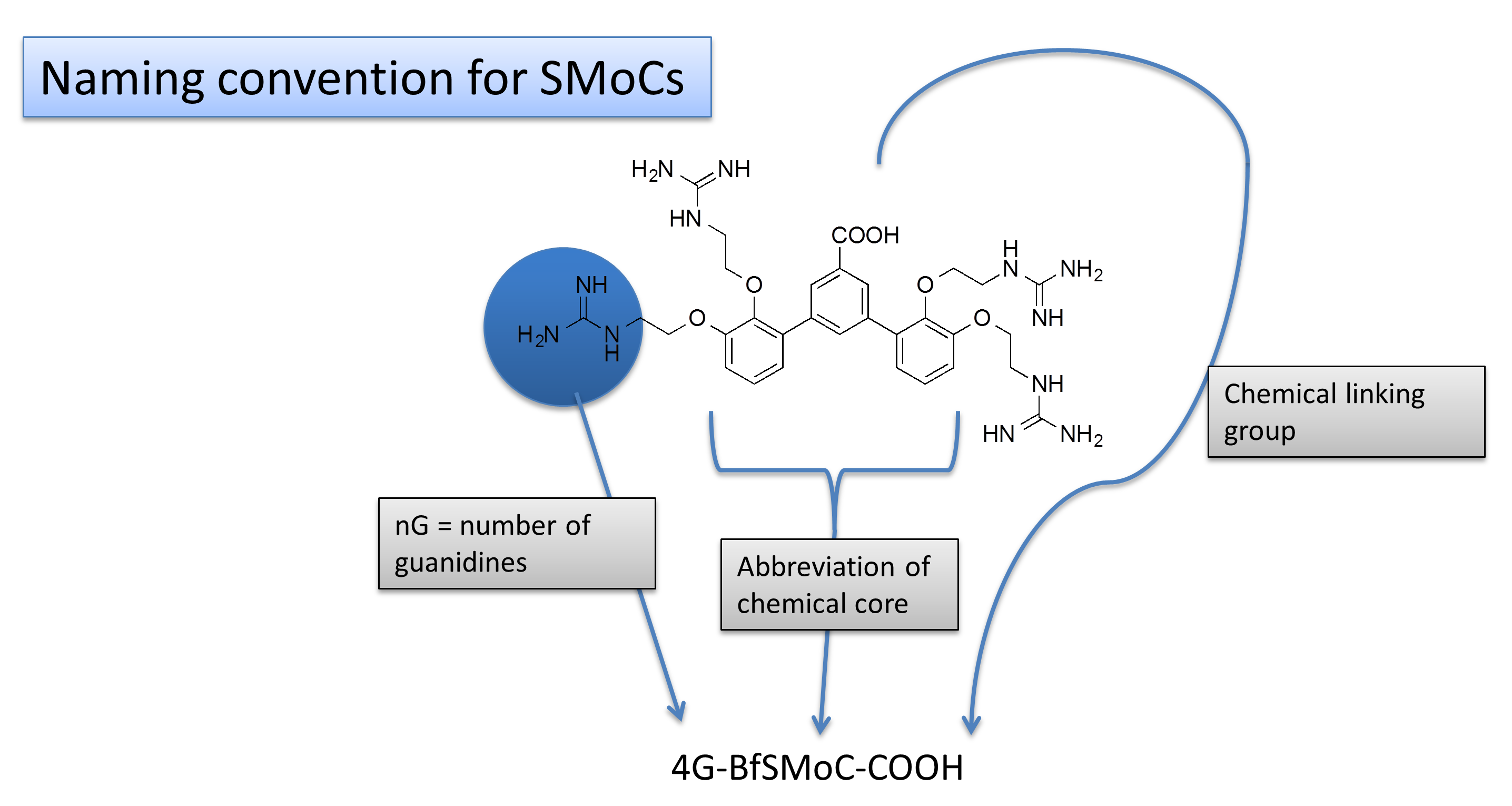


**Supplementary table S1**

Following a request from a reviewer we have included all the ^1^H NMRs for the compounds used in this study.

| **ID** | **Structure** | **Short name** | **^1^H NMR** | **Reference** |
| --- | --- | --- | --- | --- |
| **1** |  | 4G-SMoC-SSPy | ^1^H-NMR (MeOH-d4): δ2.73 (t, J = 6.6 Hz, 2H), 3.10 (t, J = 6.6Hz, 2H), 3.23-3.30 (m, 4H), 3.62-3.67 (m, 4H), 3.88 (m, 2H), 3.96 (m, 2H), 4.22 (m, 4H), 4.47 (s, 2H), 6.87 (dd, J = 7.4, 1.7 Hz, 1H), 7.02 (d, J = 7.9 Hz, 1H), 7.08-7.24 (m, 4H), 7.76-7.80 (m, 2H), 8.38 (d, J = 4.7 Hz, 1H), 8.70 (br t, 1H). | [^1^](#_ENREF_1) |
| **2** | 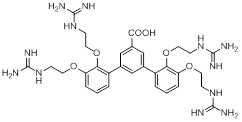 | 4G-BfSMoC | ^1^H NMR (600 MHz, D_2_O) δ 8.17 (d, *J* = 1.6 Hz, 2H), 7.86 (d, *J* = 1.1 Hz, 1H), 7.33 – 7.27 (m, 2H), 7.20 (dd, *J* = 8.3, 1.2 Hz, 2H), 7.10 (dt, *J* = 15.6, 7.8 Hz, 2H), 4.35 – 4.29 (m, 2H), 4.02 – 3.97 (m, 2H), 3.73 (t, *J* = 4.9 Hz, 2H), 3.23 – 3.18 (m, 2H). | This study |
| **12** |  | 2G-SMoC | ^1^H-NMR (500MHz, CD_3_OD, δ): 3.43 (t, *J* = 4.7 Hz, 4H, 2 x *CH_3_*NH), 4.05 (t, *J* = 4.7 Hz, 4H, 2 x *CH_3_*CH_3_NH), 7.02-7.09 (m, 4H, 4 x ArH), 7.16-7.21 (m, 2H, 2 x ArH), 7.31-7.39 (m, 2H, 2 x ArH). | [^2^](#_ENREF_2) |
| **13** |  | 3G-SMoC-SSPy | ^1^H-NMR (MeOH-d4): 2.68 (t, J = 6.8Hz, 2H), 3.07 (t, J = 6.7 Hz, 2H), 3.20 (m, 2H), 3.42 (m, 2H), 3.65 (m, 2H), 3.90 (m, 2H), 4.08 (m, 2H), 4.17 (m, 2H), 4.37 (bs, 2H), 6.86 (m, 1H), 7.05-7.29 (m, 6H), 7.73 (m, 2H), 8.37 (m, 1H). | [^3^](#_ENREF_3) |
| **8** |  | 4G-SMoC-CN | ^1^H NMR (500 MHz, MeOH) δ 7.50 (d, *J* = 8.0, 1H, aromatic), 7.20 (d, *J* = 8.0, 2H, aromatic), 7.15 (dd, *J* = 8.3, 1.6, 1H, aromatic), 6.91 (dd, *J* = 7.5, 1.6, 1H, aromatic), 4.38 (t, *J* = 5.0, 2H CH_2_), 4.24 (t, *J* = 5.2, 2H, CH_2_), 4.00 (t, *J* = 4.9, 2H, CH_2_), 3.92 (t, *J* = 4.9, 2H, CH_2_), 3.68 (dt, *J* = 9.8, 5.1, 4H, 2 x CH_2_), 3.28 (dd, *J* = 9.6, 4.7, 4H, 2 x CH_2_). | This study |
| **14** |  | 6G-SMoC | ^1^H-NMR (500 MHz, D_2_O, δ): 2.99–3.17 (m, 8H), 3.46–3.60 (m, 4H), 3.82 (dd, *J* = 34.6, 30.1 Hz, 8H), 4.05–4.23 (m, 4H), 6.77–6.90 (m, 2H), 6.95–7.22 (m, 6H). | [^2^](#_ENREF_2) |

1. Okuyama, M.; Laman, H.; Kingsbury, S. R.; Visintin, C.; Leo, E.; Eward, K. L.; Stoeber, K.; Boshoff, C.; Williams, G. H.; Selwood, D. L. Small-molecule mimics of an [alpha]-helix for efficient transport of proteins into cells. *Nature Methods* **2007,** 4, 153-159.

2. Gooding, M.; Tudzarova, S.; Worthington, R. J.; Kingsbury, S. R.; Rebstock, A. S.; Dube, H.; Simone, M. I.; Visintin, C.; Lagos, D.; Quesada, J. M.; Laman, H.; Boshoff, C.; Williams, G. H.; Stoeber, K.; Selwood, D. L. Exploring the interaction between siRNA and the SMoC biomolecule transporters: implications for small molecule-mediated delivery of siRNA. *Chem Biol Drug Des* **2012,** 79, 9-21.

3. Rebstock, A. S.; Visintin, C.; Leo, E.; Posada, C. G.; Kingsbury, S. R.; Williams, G. H.; Stoeber, K.; Selwood, D. L. Modular assembly using sequential palladium coupling gives easy access to the SMoC class of cellular transporters. *ChemBioChem* **2008,** 9, 1787-1796.
